# Wrangling distributed computing for high-throughput environmental science: An introduction to HTCondor

**DOI:** 10.1371/journal.pcbi.1006468

**Published:** 2018-10-03

**Authors:** Richard A. Erickson, Michael N. Fienen, S. Grace McCalla, Emily L. Weiser, Melvin L. Bower, Jonathan M. Knudson, Greg Thain

**Affiliations:** 1 Upper Midwest Environmental Sciences Center, United States Geological Survey, La Crosse, Wisconsin, United States of America; 2 Wisconsin Water Science Center, United States Geological Survey, Middelton, Wisconsin, United States of America; 3 Department of Computer Science, University of Wisconsin–Madison, Madison, Winconsin, United States of America; Genome Quebec, CANADA

## Abstract

Biologists and environmental scientists now routinely solve computational problems that were unimaginable a generation ago. Examples include processing geospatial data, analyzing -omics data, and running large-scale simulations. Conventional desktop computing cannot handle these tasks when they are large, and high-performance computing is not always available nor the most appropriate solution for all computationally intense problems. High-throughput computing (HTC) is one method for handling computationally intense research. In contrast to high-performance computing, which uses a single "supercomputer," HTC can distribute tasks over many computers (e.g., idle desktop computers, dedicated servers, or cloud-based resources). HTC facilities exist at many academic and government institutes and are relatively easy to create from commodity hardware. Additionally, consortia such as Open Science Grid facilitate HTC, and commercial entities sell cloud-based solutions for researchers who lack HTC at their institution. We provide an introduction to HTC for biologists and environmental scientists. Our examples from biology and the environmental sciences use HTCondor, an open source HTC system.

## Author summary

Computational biology often requires processing large amounts of data, running many simulations, or other computationally intensive tasks. In this hybrid primer/tutorial, we describe how high-throughput computing (HTC) can be used to solve these problems. First, we present an overview of high-throughput computing. Second, we describe how to break jobs down so that they can run with HTC. Third, we describe how to use HTCondor software as a method for HTC. Fourth, we describe how HTCondor may be applied to other situations and a series of online tutorials.

*This is a* PLOS Computational Biology *Education paper*.

## Introduction

### Motivation: Large computing needs and biology

The life and environmental sciences are becoming computationally intense disciplines [1]. Individual studies can generate gigabytes and even petabytes of data [2, 3]. Examples include -omics data [2, 4], high-resolution imagery [5], lidar [6], and real-time monitoring data [3]. Additionally, simulation has emerged as a third branch of science to complement empirical and theoretical approaches [7]. Examples include climate projections and assessments [8, 9], responses to climate change [10, 11], and hydrological models [12, 13]. Last, scientists may need to evaluate models using computationally intensive Monte Carlo methods [14, 15].

Scientists have traditionally processed "big" data sets and run large-scale simulations using either desktop computing (and long wait times) or high-performance computing (HPC; with higher overhead costs in a central location). These "supercomputers" use parallel computing to locally run a simulation or process data on a single, large computer. Historically, only researchers at large institutes, national laboratories, and large universities had access to HPC systems and the funding to restructure code to run on such a computer.

### High-throughput computing

Many problems can be broken down into smaller, loosely coupled jobs (defined in Box 1) and distributed across computer pools. Examples from the environmental sciences can include simulating study designs to compare model performance and study performance [14], model calibrations [16], and processing telemetry data [17]. This alternative paradigm is called high-throughput computing (HTC) [18–20]. Originally designed as a method to "scavenge" unused computational resources (e.g., idle desktop computers) in the 1980s, HTC has emerged as a method for distributed computing that can be used with dedicated servers. HTC-based approaches require users to break a large problem into many small, independent jobs that are distributed across a pool by the HTC software. Computers then process the task in many small parts. Furthermore, this allows both the task from a specific user as well as the tasks from multiple users to be distributed and processed in parallel. HTC software automatically manages the individual jobs and is designed to be robust. Jobs may be stopped (e.g., a desktop computer is no longer idle because the owner has returned to work) or lost (e.g., a connection is lost and a job needs to be restarted). This distributed computing paradigm has led to HTC being used to run other large tasks that would be too big even for one supercomputer. Currently, many different industries and scientific fields use HTC.

One specific implementation of HTC is HTCondor. HTCondor, originally called Condor, coordinates computing resources on either dedicated machines or idle machines such as desktop computers [18]. When first developed in the 1980s, HTCondor could submit and manage thousands of jobs. Currently, HTCondor can handle more than 200,000 jobs [21]. Many different organizations use HTCondor: the Open Science Grid uses HTCondor to pool computational resources across institutions [22]; the European Organization for Nuclear Research (commonly known by its French acrynomn CERN) uses HTCondor to process high-energy physics data [23], such as finding the "God particle" [24]; Disney and DreamWorks use HTCondor to create movies (http://research.cs.wisc.edu/htcondor/images/WildLetter.pdf); the National Aeronautics and Space Administration (NASA) uses HTCondor to process telescope, atmospheric, and space data as well as run simulations [25]; the National Oceanic and Atmospheric Administration (NOAA) uses HTCondor to process weather data[26]; the Search for Extraterrestrial Intelligence (SETI) uses HTCondor to search for extraterrestrial life (http://research.cs.wisc.edu/htcondor/manual/v7.8/3_12Setting_Up.html); and the United States Geological Survey (USGS) uses HTCondor for groundwater modeling [16].

Although HTC is currently used by some environmental sciences (e.g., genomics [20]), we see the opportunity for expanded use of HTC in the environmental sciences based upon our own experiences working with researchers and resource managers. HTC could help these scientists with their current computationally intensive computing tasks. Hence, we provide an introduction to HTC for these scientists.

## HTCondor

### Overview of HTCondor

HTCondor "has enabled ordinary users to do extraordinary computing" [19]. Broadly, HTCondor is a distributed-batch computing system, and our description of HTCondor applies to other HTC systems as well. Users submit a job to HTCondor. HTCondor chooses when and where to run the job based upon the job requirement and available worker machines. HTCondor monitors the jobs' progress, and HTCondor notifies the user upon completion. Practically, this means users submit their jobs from a submit machine (see Box 1 for definitions). A job is typically a batch script on Windows or a shell script on Linux/Unix/macOS. The batch/shell script calls other programs in the workflow. A key aspect of HTCondor is that it requires no custom programming or linking to run in the cluster. This eases debugging and increases confidence of correctness, for the same job can usually be run interactively on a local machine. The submit machine submits jobs to different node machines. These node machines are computers with HTCondor installed. Node machines can be either "normal" computers (e.g., the administrative assistant's computer) or dedicated computers/servers that only HTCondor uses. Nodes have ClassAds that are like "classified advertisements" in a newspaper and communicate a machine's resources (e.g., operating system, available memory, or central processing unit [CPUs]). ClassAds are a language developed by the HTCondor project. End users will not need to change ClassAds unless they are configuring machines in the HTCondor pool. Users send submit files to the submit machine, which matches jobs to machines. Once jobs are done, HTCondor gathers up and returns output files to the user.

**Box 1. HTCondor terminology adapted from [19] and the HTCondor Manual [27]**

- **Job:** The task a user wants HTCondor to run. Sometimes the official HTCondor Documentation refers to a "job" as the large task being submitted, and other times, it refers to the jobs as the specific files being submitted.
- **Batch/shell script:** A file with instructions for a computer to execute.
- **Submit machine:** One or more computers used by HTCondor to send jobs out at the start of a run and collect jobs at the end of a run. The submit machine can also run jobs but is not required to.
- **ClassAds:** A language used by HTCondor to advertise resources on machines running HTCondor. The submit files request resources based upon the ClassAds.
- **Submit file:** A file submitted by the user to the central manager. This file tells HTCondor the job requirements and what files to run as part of the job.

Ideally, a user will not need to setup their own HTCondor pool. Many universities, government agencies, and national laboratories offer HTC facilities. Additionally, many countries have science grids available that use HTCondor (e.g., Open Science Grid in the United States [22]; GridPP in the United Kingdom [https://www.gridpp.ac.uk/]). Commercial vendors also offer HTCondor (e.g., Amazon Web Service). Last, HTCondor is free and open source, and anybody may setup their own local pool. We have included documentation outlining our installation process (AR1), but otherwise, assume users have access to their own pool.

## Materials and methods

### HTCondor Quick Start example

The HTCondor Manual provides a Quick Start Example (https://research.cs.wisc.edu/htcondor/manual/quickstart.html), also in AR1. This demonstrates HTCondor by submitting a job (sleep.bat for Windows; sleep.sh for Linux) that tells the computer to "sleep" for 6 s. The submit file (sleep.sub) instructs HTCondor where the job is located, what HTCondor should do with the executable, and what to do with the results from the executable. In this example, the "results" include a log file, an errors file, and a message file. The job is submitted using the command condor_submit sleep.sub. The job may be monitored with the condor_q command. When the job is finished running, HTCondor will update the log file and no longer show the job when condor_q is entered.

### Conceptual HTCondor example

This section describes our five steps for using HTCondor, which also apply to other HTC systems.

**Step 1: Define the computing problem.** Where are the bottlenecks and what can be broken down into small tasks? The answer may not be HTC. HPC or even code optimization or language changes may be an appropriate solution (e.g., using C++ rather than R or Python). An important consideration is if a problem is processor limited or memory limited [28]. Memory limited means that a program runs out of random access memory (RAM) memory, whereas processor limited means that calculations done by a processor constrains the program. A tradeoff exists between these two constraints. Additionally, large computing jobs can be network-intensive tasks and limited by data transfers. For these tasks, HTC may not be an ideal solution or may require special consideration to avoid network limitations. Also, some computing tasks may be able to run in parallel but cannot be tightly coupled. These jobs are ill suited for HTC and require HPC.

**Step 2: Discretize the problem into smaller jobs that may be run on HTCondor.** These small discrete problems should be "pleasantly" parallel (also known as "embarrassingly" parallel) and run independently of one another. This involves defining all input files for each job and ensuring output files do not overwrite output files from other jobs. For simulations, this involves deciding how to run simulations (e.g., should one simulation be a job or multiple simulations that share the same inputs?). Simulations are often processor limited. For data processing, breaking a task down may involve splitting data into smaller files. Data processing can often be memory limited but may be processor limited if it involves many calculations.

**Step 3: Preprocess the inputs.** For large data, this may mean breaking the data up into smaller files and having a way to identify files by some naming convention. For large simulations, for instance, this may require creating an input table of parameters in which each row corresponds to a job.

**Step 4: Run the jobs on HTCondor.** This step requires placement of a submit file and corresponding files on the central manager. Depending upon the size of the job being run, the processing might take a few minutes to run or possibly take weeks or even months.

**Step 5: Postprocess the data.** This step consists of combining the output from the previously run HTCondor jobs. Often, this step is most easily done using a scripting language such as Python or R. Pre- and postprocessing can often be done on personal computers before or after the data have been moved to the HTCondor submit machine. In some cases, both the pre- and postprocessing steps might be done using their own HTCondor submit files for large projects (i.e., breaking files or jobs down into smaller jobs to run on many machines). DAGMan is a program that is included with HTCondor capable of submitting multiple HTCondor batches (conceptually, it is a program to run and manage HTCondor submit files) and is documented in the HTCondor Manual [27].

## Sandbox code

An important and sometimes difficult step with HTCondor is "sandboxing" code. Sandboxing is necessary so that all programs will run independently and predictably across platforms. The easiest method for using HTCondor avoids sandboxing by having the same version of programs installed on local machines. For example, simple scripts that only use base R or Python might not require sandboxing. There are two downsides to this approach. First, the software must be installed on the local machines. Second, machines might not have the same version installed (e.g., Python 2.x verses 3.x).

On Windows, sandboxing can sometimes be easier because Windows executables are often independent. Thus, sandboxing the code only requires using HTCondor to send all required programs (e.g., Python.exe) with the script. All required dependencies are then locally installed with the program and deleted when HTCondor is finished running. We include examples of Sandboxing R script (AR1).

On Linux/Unix systems (including macOS), multiple options exist. In theory, programs could be sandboxed like Windows. However, Unix software architecture is built upon many small building blocks that are combined, creating intricate dependencies [29]. This can be side stepped with container programs such as Docker. Docker has the added bonus of having an HTCondor "Universe" setting that allows it to integrate well with HTCondor.

Docker works by building images (i.e., a collection of software that makes programs able to run) similar to a virtual box but with a smaller footprint (i.e., size, memory, and processor use) and takes advantage of the shared kernel across Linux platforms. An image is based upon a specified version of Linux (e.g,. Ubuntu 16.04) and has specific software installed in it (e.g., R 3.0.1 with ggplot2 2.0.1). Users may build their own image either from a Dockerfile (a script that tells Docker how to build an image) or using the command line and an existing Docker image. Many images have already been developed for commonly used programs such as Python and R, and these can often meet the needs of HTCondor users or, at the very least, serve as starting places for building your own image. We prepared a brief tutorial on Docker Images (AR1) and additional documentation may be found on the Docker homepage (https://docs.docker.com/engine/tutorials/dockerimages/).

## Examples

We have prepared examples of how to use HTCondor for scientific computing (AR1). These examples include Quick Start ("hello world"-like) examples, R examples, Python examples, and Docker examples. The repository also includes tutorials and a suggested course of study for teaching oneself HTCondor.

Additionally, we regularly use (and document) HTCondor. For example, we have simulated data sets to see how statistical trends can be recovered (AR3). This code demonstrates how to use HTCondor on Windows, including sandboxing code. We have also simulated study designs and examined how well parameters could be estimated (AR2). This example demonstrates how to use HTCondor in Linux, including Docker for sandboxing code.

Briefly, we provide a walkthrough of the statistical trends example (AR3). First, we defined our computer problem as the amount of time necessary to fit models across a wide parameter space. Second, we discretized our problem by parameter combinations (i.e., each HTCondor "job" corresponded to a set of parameter combinations). Third, we presimulated all data sets for each parameter combination and also recorded these combinations in a comma-seperated values (CSV) file. Fourth, we used a submit file to run our jobs. Fifth, we used R to combine, summarize, and plot our results.

## When not to use HTC

The previous examples all worked well with HTC because they could be discretized into smaller jobs. When jobs cannot be readily broken down, HTC may not always be the solution. However, HTCondor allows for jobs to request multiple CPUs and HTCondor can be used to manage multiple HPC jobs across HPC resources. As a concrete example, running a computationally intense individual-based model might require HPC resources because the model is closely connected and is not pleasantly parallel. But running multiple instances of these models could be managed through HTCondor. Last, we have observed cases in which simply optimizing code removed our need for HTC or HPC.

## Additional resources

Learning how to use HTCondor can be overwhelming because of the amount of documentation. The manual for the stable version (8.6.9) is 1,125 pages [27]! However, the project homepage (http://research.cs.wisc.edu/htcondor/) contains several different resources that are more accessible than the manual. Talks from previous HTCondor Weeks are one resource for learning about HTCondor that include many different introductions and getting-started tutorials. Fienen and Hunt [15] also provided an introduction to HTC and HTCondor for groundwater modeling, and this may be helpful for environmental scientists. Researchers at major universities may not be aware of access they already have to HPC and HTC computing facilities. Finally, computer scientists may find an environmental or ecological computational problem an interesting computer science problem if life scientists seek out collaborations with them (personal communication, R.A. Erickson). Furthermore, working with software engineers can help to improve environmental modeling [30].

### AR1: Git repository of HTCondor tutorial

https://my.usgs.gov/bitbucket/projects/CDI/repos/hunting_invasive_species_with_htcondor/

### AR2: Git repository of simulated study design

This Git repository contains an example of how to use HTCondor to simulate and recover a study design using R, RStan, and Docker: https://my.usgs.gov/bitbucket/users/rerickson_usgs.gov/repos/edna-sampling-design/browse.

### AR3: Git repository for trend comparison

Git repository contains an example of how to use HTCondor to compare trend estimation R, MARSS, and Docker: https://my.usgs.gov/bitbucket/projects/UMESC/repos/ltrm-trend_comparison/browse.

## Conclusion

Biology and the environmental sciences have become computationally intense. HTC is an important framework for dealing with computationally intense problems. We provided an introduction to HTC and an overview of using HTCondor. Our specific five steps were 1) define the computational problem and confirm that HTC is the solution, 2) determine how a problem can be broken down into small discrete jobs and can be identified by a unique number, 3) preprocess inputs into small jobs that can be distributed, 4) run the job using HTC (e.g., HTCondor), and 5) postprocess the data and combine the results from the many distributed jobs.
